# The association between sport-technology use and kinesiophobia among people with disabilities: serial multiple mediation of health locus of control and self-efficacy

**DOI:** 10.3389/fpsyg.2026.1802772

**Published:** 2026-07-14

**Authors:** Jian Jiao

**Affiliations:** School of Physical Education and Sports, Shenzhen University of Information Technology, Shenzhen, China

**Keywords:** exercise self-efficacy, health locus of control, kinesiophobia, serial multiple mediation, sport-technology use

## Abstract

**Background:**

In the fear-avoidance model, kinesiophobia is a critical affective barrier that is associated with persistent activity avoidance and psychological distress among people with disabilities. While sport-technology use correlates with lower levels of kinesiophobia, the indirect pathways that may explain this relationship remain underexplored, especially in individuals who are already physically active and engaged with technology. This study examined how sport-technology use relates to kinesiophobia in a specific subpopulation of adults with sensory or physical disabilities, and explored the independent and serial associative roles of health locus of control and self-efficacy in this relationship.

**Methods:**

A cross-sectional survey was conducted with 901 adults from China who had physical, visual, or hearing impairments, were current sport-technology users (cumulative use ≥1 month, ≥1 time/month), and had participated in physical activity within the past 6 months. Individuals with intellectual or multiple disabilities were excluded. Bootstrap analyses were used to estimate the total, direct, and indirect associations, including independent indirect associations via health locus of control and self-efficacy, as well as their serial indirect association.

**Results:**

Greater sport-technology use was associated with lower kinesiophobia (*β* = −0.268, 95% CI [−0.326, −0.209]). The total indirect association represented 33.58% of the total association. Significant indirect pathways were identified through health locus of control alone, through self-efficacy alone, and through the serial route from health locus of control to self-efficacy.

**Conclusion:**

In this group of physically active sport-technology users with sensory or physical disabilities, Greater sport-technology use predicted lower levels of kinesiophobia. This relationship was statistically mediated by independent and serial indirect effects through health locus of control and self-efficacy.

## 1 Introduction

International consensus has increasingly coalesced around safeguarding the rights of people with disabilities and promoting their physical and mental well-being. The World Health Organization (WHO), in its global report on disability and health, emphasizes that participation in physical activity is a key pathway to improving psychological status and quality of life among people with disabilities. In parallel, the International Paralympic Committee (IPC) has continued to advance adaptive sport in recent years, advocating the use of sport-technology products that may relate to lower levels of physiological and psychological barriers to sport participation. These initiatives have created broad opportunities for applying such products in disability sport and physical-activity promotion. Against this backdrop, examining the relationship between sport-technology use and kinesiophobia—and clarifying the underlying psychological mechanisms—responds to global calls for disability rights and health equity while also advancing theoretical and practical innovation in disability sport and health.

Physical activity participation is a crucial avenue for fostering social inclusion and enhancing quality of life for people with disabilities. However, kinesiophobia, as a salient affective barrier, remains a major constraint on engagement in physical activity. Prior research suggests that kinesiophobia is closely associated with functional impairments, previous exercise-related traumatic experiences, and diminished health locus of control. This fear of movement not only undermines and adherence to exercise but may also exacerbate negative psychological states, thereby impeding physical and psychological rehabilitation (Al Harthy et al., 2025). Sport-technology products—through precise data monitoring, gamified scenarios, and personalized adaptation—may reduce both physical access barriers and psychological stress during physical activity, thus improving exercise-related psychological outcomes (Shafizadeh and Davids, 2024). Studies on physical activity among people with disabilities further indicate that external factors such as social support and physical activity can indirectly enhance the quality of participation by strengthening psychological capital and self-efficacy (Peng et al., 2025). Moreover, evidence from health locus of control research suggests that reinforcing internal control beliefs may promote the development of health behaviors partly by enhancing self-efficacy (Li et al., 2026).

Nevertheless, existing studies have predominantly examined single-mediator pathways and have rarely provided a systematic test of whether sport-technology use is negatively associated with kinesiophobia via the serial mediation of health locus of control and self-efficacy. To address this gap, the present study investigates the chain-mediating mechanism linking sport-technology use to kinesiophobia among people with disabilities. By elucidating the potential psychological intervention effects of sport-technology products, this work offers actionable implications for product optimization and the design of physical-activity interventions for people with disabilities. The findings are expected to support efforts to alleviate kinesiophobia, enhance physical activity participation capacity, and ultimately facilitate health promotion and social inclusion.

## 2 Literature review and hypotheses

### 2.1 Sport-technology use and kinesiophobia among people with disabilities

Sport-technology products, emerging from the deep integration of modern technologies and the sport/exercise domain, are increasingly being adopted in rehabilitation and everyday physical activity contexts among people with disabilities. Owing to their assistive, intelligent, and personalized features, these products offer novel technological pathways to address barriers to physical activity participation in this population. Kinesiophobia among people with disabilities largely stems from perceived uncontrollability during physical activity, which can trigger anxiety and avoidance behaviors and may impose persistent constraints on rehabilitation and health behavior engagement (Lundberg and Archer, 2022). By leveraging intelligent technologies, sport-technology products can enhance perceived controllability of the exercise process. For example, smart exercise devices can monitor activity in real time and provide timely alerts for potential risks (e.g., abnormal heart rate or movement deviations), enabling users to adjust their exercise status promptly and thereby be negatively associated with the likelihood of adverse events (De Fazio et al., 2023).

In addition, sport-technology use may attenuate kinesiophobia through multiple pathways. First, assistive functions can lower the threshold for participation by reducing exercise difficulties caused by functional impairments, thereby preventing fear responses from being reinforced by repeated frustration or failure experiences (Willingham et al., 2024). Second, research on community-based fitness and sport participation among people with disabilities highlights the importance of social support strategies—such as peer support, professional support, motivational support, and organized social activities—in promoting exercise adherence and positive participation experiences (Kennedy et al., 2022). When users can exchange experiences, share achievements, and receive data-driven feedback via digital platforms, their progress becomes more “visible,” which may be positively correlated with exercise motivation and further alleviate kinesiophobia.

Taken together, sport-technology use may be negatively associated with kinesiophobia among people with disabilities by increasing perceived controllability during exercise, lowering perceived risk, and providing positive reinforcement and emotional support. Therefore, the present study proposes the following hypothesis:

*H1*: Sport-technology use is significantly negatively associated with kinesiophobia among people with disabilities, and is negatively associated with kinesiophobia.

### 2.2 The mediating correlate of health locus of control

Health locus of control, a central psychological construct in health self-management, refers to individuals’ perceived capacity to appraise, regulate, and control their health status, health-related behaviors, and health outcomes. Its level is closely associated with physical and mental well-being, quality of life, and social participation among people with disabilities (Hajek and König, 2017). The monitoring and feedback functions of sport-technology products enable people with disabilities to obtain health indicators and exercise-related data in a clear and accurate manner (e.g., heart rate, exercise duration, and rehabilitation progress). By reducing health-information asymmetry stemming from functional impairments, these products allow users to perceive changes in their health status more directly, thereby strengthening perceived control over health (Latif et al., 2024). In addition, the personalized and user-friendly nature of sports-technology products can be tailored to diverse functional limitations, providing exercise and rehabilitation programs that can be autonomously operated and flexibly adjusted. Such features may reduce dependence on others and enable users to independently determine exercise timing, intensity, and rehabilitation goals, gradually enhancing autonomy in health management and reinforcing their capacity to regulate and control health behaviors (Huang et al., 2025). Over time, sustained use of sport-technology products may facilitate the development of regular exercise habits and promote functional improvement and better health outcomes; in turn, positive health changes can further be positively correlated with confidence in health management and elevate health locus of control (Peng et al., 2021).

From a cognitive–behavioral perspective, emotional reactions and behavioral tendencies are not determined directly by events themselves but are mediated by individuals’ cognitive appraisals, within which perceived control constitutes a key determinant shaping appraisal direction and outcomes (Pankowski et al., 2023). For people with disabilities, uncertainty in exercise contexts may heighten concerns about physical limits and potential injuries, and differences in health locus of control can be associated with divergent appraisals of exercise-related situations. Individuals with a higher health locus of control are more likely to understand their current health condition and functional boundaries, evaluate potential health risks more rationally, and believe that health threats can be prevented or managed through appropriate exercise modalities and intensity regulation. Such control-related cognitions are negatively associated with the expectation of negative outcomes, thereby decreasing anxiety and avoidance tendencies and ultimately alleviating kinesiophobia (Özden et al., 2025). Moreover, individuals with higher health locus of control may better accept functional impairments and affirm their health-related self-worth, which prevents them from negating their exercise capability due to physical limitations; consequently, they may be more willing to attempt physical activity and confront challenges, further reducing kinesiophobia. Accordingly, the present study proposes the following hypotheses:

*H2*: Sport-technology use is a positive correlate of health locus of control among people with disabilities.

*H3*: Health locus of control is inversely related to kinesiophobia among people with disabilities.

*H4*: The association between sport-technology use and kinesiophobia among people with disabilities is is partially mediated by health locus of control.

### 2.3 The mediating correlate of self-efficacy

Self-efficacy, proposed by Bandura within social cognitive theory, refers to an individual’s confidence in their capability to use the skills they possess to accomplish a specific task and is considered a core indicator of psychological state and capability beliefs. Through intelligent design and personalized adaptation, sport-technology products can tailor exercise programs to individuals’ health status and functional capacity, may lower task difficulty and reduce participation barriers, and reinforce recognition of one’s exercise capabilities, thereby gradually enhancing self-efficacy (Lawrason et al., 2022). Features such as online communities and video-guided modules may further help people with disabilities recognize that they are able to complete exercise tasks and achieve personal breakthroughs, correcting maladaptive beliefs such as “I cannot exercise because of my impairment,” and indirectly strengthening efficacy beliefs (Newman et al., 2023).

Moreover, an increasing range of disability-adapted sport-technology products—from basic assistive exercise equipment to smart monitoring and coaching systems—enables users to exert greater autonomy over the exercise process and perceive their progress more clearly. Such autonomy and perceived control can enhance perceived self-worth and self-efficacy. For instance, blended programs combining online/remote exercise interventions with digital tools have been used to support physical activity participation among individuals with spinal cord injury and improve exercise-related psychological outcomes (Bombardier et al., 2021). In addition, wearable activity trackers, as an important vehicle for the “self-monitoring–feedback–reinforcement” cycle, have been shown in systematic reviews to promote physical activity and yield health-related benefits, providing visible evidence of progress that may facilitate the development of self-efficacy (Froehlich-Grobe et al., 2022). In rehabilitation contexts, interactive telerehabilitation or exergaming systems can deliver movement feedback and structured training, linked to lower levels of reliance on external assistance, thereby helping users accumulate mastery experiences and build confidence (Chen et al., 2021).

For people with disabilities, engaging in physical activity often involves multiple challenges, including increased motor difficulty due to functional limitations, elevated injury risk, and pressure from social evaluation. Individuals with higher self-efficacy are more likely to draw on prior mastery experiences, vicarious experiences, and social support to evaluate their exercise capabilities objectively and believe they can cope effectively with potential difficulties and risks (e.g., regulating exercise intensity, preventing injury, and adapting to unexpected situations). Such positive capability beliefs foster more rational appraisals of exercise contexts and are associated with fewer irrational fear responses. Prior research has documented close associations between exercise-related fear, self-efficacy, and physical activity levels: individuals with higher self-efficacy typically report greater physical activity participation and lower fear, which in turn is associated with lower levels of kinesiophobia and greater engagement (Zhang et al., 2023). This relationship may be particularly salient among people with disabilities, for whom functional constraints further increase the difficulty and risk of physical activity, amplifying the predictive and protective correlate of self-efficacy. In addition, kinesiophobia in this population is often linked to frustration experiences during exercise and negative social evaluations; self-efficacy may buffer these associations by enabling individuals to interpret setbacks as part of skill development rather than as evidence of incompetence and to resist discouraging social feedback, thereby sustaining motivation and reducing fear. Accordingly, the present study proposes the following hypotheses:

*H5*: Sport-technology use is positively associated with self-efficacy among people with disabilities.

*H6*: Self-efficacy is negatively associated with kinesiophobia among people with disabilities.

*H7*: The association between sport-technology use and kinesiophobia among people with disabilities is partially mediated by self-efficacy.

### 2.4 Serial mediation of health locus of control and self-efficacy

Health locus of control and self-efficacy both involve individuals’ subjective appraisals of their own capabilities and the external environment. Health locus of control can be conceptualized as a health-specific constellation of control beliefs and capability beliefs, whereas self-efficacy reflects confidence in one’s ability to execute behaviors required to achieve specific goals. In research on health behavior change, these two constructs are frequently incorporated within the same explanatory framework and show robust associations (Stewart-Knox et al., 2025). Because health is foundational to survival and development, health locus of control is likely to shape broader efficacy beliefs relevant to health-related action. Individuals with a higher health locus of control tend to believe more strongly that their behaviors can effectively regulate health status, enabling them to proactively avoid health risks and cope with health challenges. Such experiences of “controllability and effectiveness” can increase engagement and persistence in health behavior change and have been linked to more adaptive response patterns across a range of health behavior interventions (Mozafari et al., 2024).

A growing body of evidence indicates that individuals with higher health locus of control typically demonstrate stronger enactment of health behaviors and greater self-management capacity. In chronic disease management contexts, health locus of control has been shown to play a pivotal correlate within pathways such as “empowerment → self-efficacy → self-management behaviors,” thereby influencing efficacy beliefs and sustained adherence to health behaviors (Wang et al., 2022). Research on treatment adherence and long-term health management further suggests that strengthening internal control beliefs together with self-efficacy can promote persistence in health-related behaviors and adherence to treatment regimens (Buster and Ozsaker, 2022), and self-efficacy itself is positively associated with self-management adherence among individuals with chronic conditions. From a cognitive processing perspective, individuals with a higher health locus of control are more inclined to interpret health outcomes through internal and modifiable explanations; this cognitive orientation is associated with higher self-efficacy and greater investment in health-protective behaviors (Zhang et al., 2022). Conversely, external control attributions combined with low self-efficacy may predispose individuals to negative affect and psychological distress, providing convergent support for the proposed chain of “control beliefs → efficacy beliefs → adaptive outcomes” (Haywood and Mason, 2023). Evidence specific to people with disabilities further indicates that the association between health control beliefs and life satisfaction can be a mediating pathway through self-efficacy, and this mechanism appears particularly informative in contexts involving mobility limitations (Rogowska et al., 2020).

Integrating the above evidence, we posit a sequential pathway of “health locus of control → self-efficacy” based on two core theoretical premises: first, health locus of control reflects a domain-general, stable belief about one’s ability to control health outcomes, which serves as a cognitive foundation for the formation of domain-specific self-efficacy beliefs related to exercise behavior; second, social cognitive theory posits that generalized control beliefs precede and shape situation-specific efficacy expectations, rather than the reverse.

That said, we acknowledge an alternative, reversed relational orientation: sport-technology use may first enhance exercise self-efficacy, which in turn strengthens general health locus of control, forming a serial pathway of “self-efficacy → health locus of control”. We test this alternative model in subsequent analyses to empirically verify the rationality of our hypothesized sequential order. Beyond this, we also note other divergent relational frameworks, such as parallel mediation models where health locus of control and self-efficacy operate independently without sequential transmission, and moderated mediation models where disability type or functional impairment severity may shape the strength of the mediating pathways; these alternative frameworks are further addressed in the discussion section. Accordingly, the present study proposes the following hypotheses:

*H8*: Health locus of control is positively associated with self-efficacy among people with disabilities.

*H9*: The association between sport-technology use and kinesiophobia among people with disabilities is partially mediated by the serial pathway via health locus of control and self-efficacy.

**Figure 1:**
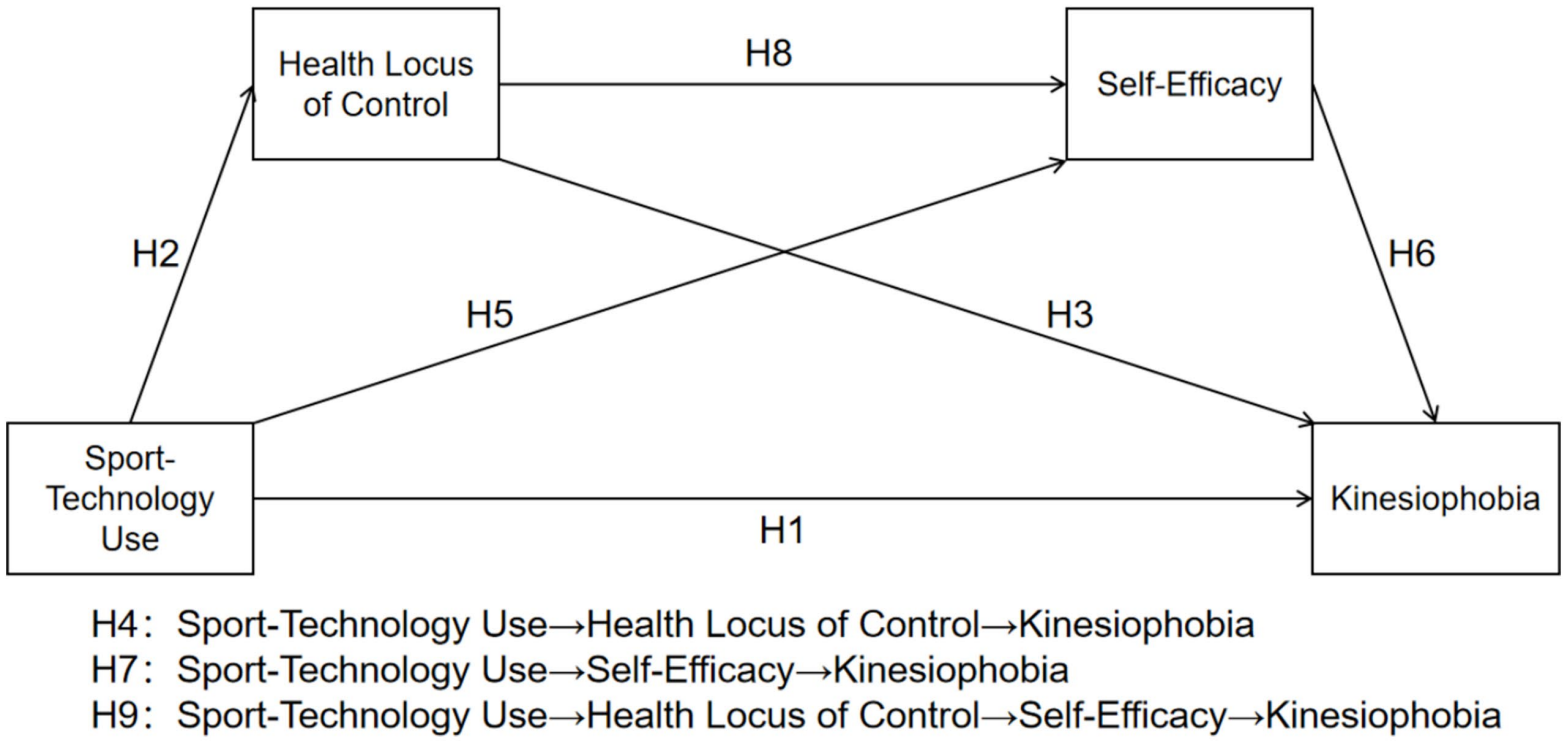
Theoretical model.

Figure 1 depicts the hypothesized pathways.

## 3 Methods

### 3.1 Participants

This study targeted people with disabilities who use sport-technology products. A multi-stage stratified convenience sampling strategy combined with snowball sampling was employed to balance sample representativeness and feasibility.

In the first stage (stratified sampling), participants were stratified by disability type based on the Classification and Grading Criteria for Persons with Disabilities and grouped into three categories: physical impairment, visual impairment, and hearing impairment. Individuals with mental disabilities, intellectual disabilities, and those with multiple disabilities who were unable to independently complete the questionnaire or lacked the ability to engage in physical activity were excluded. This approach ensured coverage across disability types and was linked to lower levels of potential bias caused by over-representation of a single category. Participants were further stratified by region, with recruitment conducted across three major regions of China (eastern, central, and western), selecting 3–4 cities within each region.

In the second stage (convenience sampling combined with snowball sampling), eligible participants within each stratum were recruited through multiple channels, including (a) local Disabled Persons’ Federations (DPFs), (b) disability-focused public welfare organizations, (c) sport rehabilitation institutions and offline experience sites for sport-technology products, and (d) online communities (e.g., WeChat groups for disability sports, official accounts of DPFs). Recruited participants were also invited, on a voluntary basis, to refer other eligible individuals within their social networks to expand coverage and mitigate the limitations of single-channel recruitment.

To provide transparency regarding participant sources, the final sample of 901 valid respondents was distributed across recruitment channels as follows: 286 (31.7%) from local DPFs, 194 (21.5%) from disability-focused public welfare organizations, 168 (18.6%) from sport rehabilitation institutions/offline experience sites, 153 (17.0%) from online communities, and 100 (11.1%) through snowball referrals. The snowball subsample originated from 35 initial seeds who were recruited from the other four channels, ensuring diversity of referral starting points.

To minimize selection bias, we implemented several procedural safeguards. First, we employed a multi-source recruitment strategy that combined institution-based channels (DPFs, rehabilitation centers) with community-based and online channels, reducing the risk of over-representing any single type of user (e.g., only highly motivated or technologically savvy individuals). Second, within each disability type and region, we set approximate quota targets based on local disability prevalence statistics from the China Disabled Persons’ Federation, and we monitored enrolment ratios weekly to avoid disproportionate contributions from any channel. Third, we systematically compared key demographic and study variables across recruitment channels using ANOVA (for continuous variables) and chi-square tests (for categorical variables). No statistically significant differences were found in age, disability type distribution, sport-technology use score, or kinesiophobia score across channels (all *p* > 0.05), suggesting that channel-specific selection bias was not a serious concern. Fourth, for the snowball component, we limited the referral chain length to a maximum of three waves and instructed seeds to refer only individuals who met the inclusion criteria and who were not already known to be enrolled, thereby reducing clustering bias. Finally, we conducted sensitivity analyses by excluding the snowball subsample (n = 100) and re-running the main mediation models; the results remained largely comparable, suggesting that the inclusion of snowball referrals introduced no major bias into the findings.

Regarding incentives, participants who completed the questionnaire were offered a small monetary reward of RMB 2 in the form of a cash voucher or an instant WeChat red packet (hongbao). For offline participants who preferred non-monetary incentives, we offered a small rehabilitation-themed gift (e.g., a portable hand grip strengthener or a set of elastic exercise bands) of equivalent retail value (RMB 5–10). The incentive amount was chosen to be sufficient to acknowledge participants’ time and effort (the questionnaire took approximately 15–20 min to complete) without being large enough to unduly influence response authenticity or attract respondents who did not genuinely meet the inclusion criteria. All incentives were provided after the completed questionnaire was screened for basic completeness and logical consistency. Small incentives were provided to improve the response rate, and a 20-min time limit was set to reduce careless responding. Data collection lasted 4 weeks. Online responses were monitored regularly, and offline questionnaires were collected on a rolling basis. All returned questionnaires were screened, invalid responses were removed, and valid questionnaires were coded.

In total, 1,000 questionnaires were distributed (online and offline), 972 were returned, and 61 were excluded as invalid. The final sample comprised 901 valid questionnaires, yielding an effective response rate of 90.1%. The distribution of demographic characteristics is presented in Table 1.

**Table 1:** Demographic characteristics of the participants.

Variable	Category	Frequency	Percentage
Region	Eastern	314	34.9
	Central	291	32.3
	Western	296	32.9
Age	20 ~ 30	221	24.5
	31 ~ 40	244	27.1
	41 ~ 50	230	25.5
	51 ~ 60	206	22.9
Residence	Rural	442	49.1
	Urban	459	50.9
Disability type	Physical impairment	311	34.5
	Visual impairment	304	33.7
	Hearing impairment	286	31.7

It is crucial to explicitly acknowledge the specific nature of the final sample (N = 901). By design, the inclusion criteria deliberately focused on a sub-population of individuals with disabilities who: (a) have used sport-technology products for at least 1 month (≥1 time/month); and (b) have been physically active within the past 6 months. This design choice was essential for ensuring that participants could validly report on the core constructs of interest (technology use and exercise-related fear). However, it inherently excludes those who are technologically naïve, physically inactive, or have other disability types (e.g., intellectual disabilities, multiple disabilities).

Therefore, the findings of this study are statistically generalizable only to the defined sub-population: Chinese adults with physical, visual, or hearing impairments who are current, regular users of sport-technology products and maintain a basic level of physical activity (at least some participation in the past 6 months). Extrapolating these results to the entire population of people with disabilities—particularly to those who are inactive, have no access to or interest in sport-technology, or have intellectual/multiple disabilities—should be done with extreme caution and requires further empirical validation. This limitation is further addressed in the Discussion section.

### 3.2 Measures

#### 3.2.1 Sport-technology use scale

Sport-technology use was assessed using a scale developed by Li et al. (2026) and validated in the Chinese sociocultural context. The scale comprises three dimensions—behavioral frequency, functional depth, and sustained dependence—with 8 items in total. Items are rated on a 5-point Likert scale, yielding total scores ranging from 8 to 40; higher scores indicate greater sport-technology use. In the present study, the scale demonstrated good construct validity (KMO = 0.877; Bartlett’s test of sphericity = 1228.698, *p* < 0.001) and acceptable internal consistency (Cronbach’s *α* = 0.771).

#### 3.2.2 Tampa scale of Kinesiophobia

Kinesiophobia was measured using the Tampa Scale of Kinesiophobia (TSK), originally developed by Kori (1990) and translated into Chinese by Wen (2012). The Chinese version has been validated in prior research. The TSK contains 17 items rated on a 5-point Likert scale; items 4, 8, 12, and 16 are reverse-scored. Total scores range from 17 to 85, with higher scores indicating greater kinesiophobia. In this study, the TSK exhibited excellent construct validity (KMO = 0.956; Bartlett’s test = 3539.306, *p* < 0.001) and good reliability (Cronbach’s α = 0.875).

#### 3.2.3 Multidimensional health locus of control scale

Health locus of control was assessed using the Multidimensional Health Locus of Control (MHLC) Scale developed by Wallston et al. (1978). The MHLC includes three subscales—Internal, Powerful Others, and Chance—with 6 items per subscale (18 items in total), rated on a 5-point Likert scale. Each subscale score ranges from 6 to 30, with higher scores indicating a stronger orientation toward the corresponding locus of control. Consistent with the focus of the present study on perceived personal control over health, the Internal subscale score was used as the indicator of health locus of control in subsequent analyses. This selection is further supported by both theoretical and empirical grounds: Theoretically, the Internal subscale has a perfect conceptual match with our hypothesized mediation pathway of “sport-technology use → strengthened autonomous health control beliefs → enhanced self-efficacy → reduced kinesiophobia”. Empirically, this subscale is a widely validated core indicator for measuring individual health control beliefs in exercise psychology research among people with disabilities, and is highly compatible with the population characteristics and research questions of this study. The MHLC demonstrated excellent construct validity (KMO = 0.957; Bartlett’s test = 3750.113, *p* < 0.001) and good internal consistency (Cronbach’s *α* = 0.880).

#### 3.2.4 Self-efficacy scale

Self-efficacy was measured using the General Self-Efficacy Scale (Liu et al., 2023). The scale comprises 10 items rated on a 5-point Likert scale, with total scores ranging from 10 to 50; higher scores indicate higher self-efficacy. We acknowledge the conceptual distinction between general self-efficacy and exercise-specific self-efficacy: the latter is more proximal to our exercise-specific outcome of kinesiophobia. We selected GSES for two core reasons aligned with our study design: (1) Theoretical coherence: it matches the domain-general level of our health locus of control measure, ensuring logical consistency of the serial mediation pathway; (2) Population adaptability: it has well-established reliability and measurement equivalence across the physical, visual, and hearing impairment groups in our sample, which most exercise-specific scales lack for this heterogeneous population. In the present study, the scale showed good construct validity (KMO = 0.901; Bartlett’s test = 1550.189, *p* < 0.001) and acceptable internal consistency (Cronbach’s *α* = 0.793).

### 3.3 Data processing and statistical analysis

All statistical analyses were performed using SPSS 27.0 and PROCESS 4.0. First, the dataset was coded and screened (e.g., variable classification, data transformation, and computation of composite scores). Second, common method bias was assessed using Harman’s single-factor test. Third, scale reliability and construct validity were evaluated using Cronbach’s α, KMO, and Bartlett’s test of sphericity. Fourth, descriptive statistics and Pearson correlation analyses were conducted to examine bivariate associations among all study variables, including demographic and disability-related covariates. Hierarchical multiple linear regression analyses were then performed to test the net predictive effects of sport-technology use, health locus of control, and self-efficacy on kinesiophobia, after controlling for *a priori* selected covariates: age, residence (urban/rural), region (eastern/central/western China), and disability type. Specifically, Model 1 only included the covariates to examine their explanatory power for kinesiophobia; Model 2 added the core independent variable and mediators on the basis of Model 1, to test the independent predictive effects of core variables after eliminating the confounding influence of covariates. Variance inflation factor (VIF) and tolerance statistics were calculated to diagnose multicollinearity in all regression models.

Finally, the serial multiple mediation effects were tested using the bootstrapping procedure in PROCESS 4.0 Macro (Model 6) for SPSS, with the same covariates mentioned above included in the model as control variables. This procedure can adjust for the potential influence of measured confounding variables, thereby mitigating bias and obtaining more robust estimation of mediation effects. We estimated the direct effect of sport-technology use on kinesiophobia, as well as the total indirect effect and three specific indirect effects: (1) the independent mediating pathway via health locus of control; (2) the independent mediating pathway via self-efficacy; (3) the serial mediating pathway via “health locus of control → self-efficacy”. All effect estimations were based on 5,000 bootstrap samples, and 95% bias-corrected confidence intervals (CIs) were reported. An effect was considered statistically significant when its 95% CI did not contain zero. Indirect effects were considered statistically significant when the CI did not include zero.

Given the cross-sectional design of this study, all uses of the term ‘prediction’ in the results section refer exclusively to statistical prediction derived from regression models, and do not imply temporal sequence or causal relationships.

## 4 Results

### 4.1 Common method Bias test

To minimize potential common method bias (CMB), this study adopted a systematic approach combining ex ante procedural remedies and ex post statistical tests. During questionnaire design and data collection, anonymity was ensured and participants were informed that the data would be used solely for academic research and kept strictly confidential, thereby reducing social desirability bias. Items measuring different variables were presented in a randomized order to prevent systematic response patterns. Clear instructions were provided, and a 20-min time limit was set; small incentives were offered to encourage careful responding. In addition, questionnaires were distributed through authoritative channels (e.g., Disabled Persons’ Federations) to enhance response authenticity.

After data collection, Harman’s single-factor test was conducted as an ex post check. The first unrotated factor accounted for 16.017% of the total variance, which is below the commonly used 40% threshold, indicating that CMB was unlikely to be a serious concern. Moreover, variance inflation factors (VIFs) from multiple linear regression analyses were examined as an additional diagnostic indicator. The VIF values for sport-technology use, health locus of control, and self-efficacy were 1.143, 1.101, and 1.098, respectively—well below the conventional cutoff of 10—and all tolerance values exceeded 0.10. These results suggest no serious multicollinearity among the predictors and provide further indirect support that common method bias did not substantially distort the findings.

Overall, by implementing both procedural controls and statistical diagnostics (Harman’s single-factor test and VIF/tolerance checks), this study effectively mitigated the potential influence of common method bias, supporting the adequacy of data quality for subsequent analyses and the robustness of the results.

### 4.2 Correlations among study variables

As shown in Table 2, the four core variables exhibited relatively concentrated distributions in descriptive statistics. The mean score for sport-technology use was 3.02 (SD = 0.73), kinesiophobia was 2.96 (SD = 0.68), health locus of control was 3.04 (SD = 0.68), and self-efficacy was 2.97 (SD = 0.71). Standard deviations ranged from 0.68 to 0.73, indicating a relatively balanced dispersion of responses and stable distributions across variables.

**Table 2:** Correlations among the study variables.

Variable	Mean	SD	STU	KPH	HLOC	SE
STU	3.02	0.73	1			
KPH	2.96	0.68	−0.287**	1		
HLOC	3.04	0.68	0.276**	−0.255**	1	
SE	2.97	0.71	0.271**	−0.273**	0.196**	1

*p < 0.05; **p < 0.01; ***p < 0.001, the same below. Abbreviations: STU = sport-technology use, KPH = kinesiophobia, HLOC = health locus of control, and SE = self-efficacy.

Pearson correlation analyses indicated that all associations among the main variables were statistically significant (*p* < 0.01). Sport-technology use showed a significant negative correlation with kinesiophobia (*r* = −0.287), suggesting that higher levels of sport-technology use were associated with lower kinesiophobia among people with disabilities. Sport-technology use was significantly positively correlated with health locus of control (*r* = 0.276) and self-efficacy (*r* = 0.271), indicating that greater sport-technology use was associated with stronger perceived control over health and higher self-efficacy. Both health locus of control (*r* = −0.255) and self-efficacy (*r* = −0.273) were significantly negatively correlated with kinesiophobia, suggesting that stronger health control beliefs and greater self-efficacy are linked to lower kinesiophobia. In addition, Health locus of control had a significant positive correlation with self-efficacy (*r* = 0.196), providing a correlational prerequisite for testing the proposed serial mediation pathway between sport-technology use and kinesiophobia.

To examine the effects of sport-technology use, health locus of control, and self-efficacy on kinesiophobia among people with disabilities, a multiple linear regression model was estimated. The results are presented in Table 3. The intercept was statistically significant (*B* = 4.545, SE = 0.129, t = 35.167, 95% CI [4.291, 4.798]), as its confidence interval did not include zero.

**Table 3:** Results of the multiple linear regression analysis.

Variable	B	SE	β	t	LLCL	ULCL	Tol.	VIF
Constant	4.545	0.129		35.167	4.291	4.798		
STU	−0.177	0.031	−0.190	−5.76	−0.237	−0.117	0.875	1.143
HLOC	−0.168	0.033	−0.166	−5.131	−0.232	−0.104	0.908	1.101
SE	−0.182	0.031	−0.189	−5.859	−0.243	−0.121	0.91	1.098
*R*^2^_adj_				0.146				
F				52.135^***^				

In the regression model, sport-technology use was a significant negative statistical predictor of kinesiophobia (B = −0.177, SE = 0.031, *β* = −0.190, t = −5.760, 95% CI [−0.237, −0.117]). This indicates that, after controlling for the other variables in the model, greater sport-technology use was associated with lower levels of kinesiophobia. Health locus of control, the first mediator, also significantly predicted lower kinesiophobia (B = −0.168, SE = 0.033, β = −0.166, t = −5.131, 95% CI [−0.232, −0.104]), suggesting that stronger perceived control over health was linked to lower levels of kinesiophobia. Likewise, self-efficacy, the second mediator, was a significant negative predictor of kinesiophobia (B = −0.182, SE = 0.031, β = −0.189, t = −5.859, 95% CI [−0.243, −0.121]), indicating that higher self-efficacy was associated with lower kinesiophobia.

Diagnostics for multicollinearity suggested no serious concern: tolerance values ranged from 0.875 to 0.910, and variance inflation factors (VIFs) ranged from 1.098 to 1.143, all well below the conventional cutoff of 10. These results support the robustness of the regression estimates. The results of the multiple linear regression analysis showed that when using kinesiophobia as the dependent variable and including sport-technology use, health locus of control, and self-efficacy as predictive variables, the regression model as a whole was statistically significant (*F* (3, 897) = 52.135, *p* < 0.001). The model could explain 15.0% of the total variation of kinesiophobia in the population with physical disabilities. The adjusted R^2^ after correcting the number of independent variables was 0.146. In the context of health psychology and disability behavior research, an R^2^ of 0.150 can be considered a moderate effect size. Therefore, the present model demonstrates a modest but acceptable level of explanatory power for a cross-sectional study of this nature. Specifically, sport-technology use, health locus of control, and self-efficacy all showed significant negative predictive effects on kinesiophobia (all *p* < 0.01), which was completely consistent with the research hypothesis.

### 4.3 Mediation analysis

Table 4 presents the results of the mediation pathway analyses. The findings showed that the total effect of sport-technology use on kinesiophobia among people with disabilities was significant (*β* = −0.268, 95% CI [−0.326, −0.209]), indicating that greater sport-technology use was associated with lower kinesiophobia. Decomposition of the total effect revealed a significant direct effect (β = −0.178, 95% CI [−0.238, −0.117]), accounting for 66.42% of the total effect, suggesting that a substantial portion of the association operated through a direct pathway. The total indirect effect was also significant (*β* = −0.090, 95% CI [−0.117, −0.065]), explaining 33.58% of the total effect, thereby confirming the mediating pathway of health locus of control and self-efficacy.

**Table 4:** Results of the mediation pathway analysis.

Effect	Path	Effect size	Standard error	LLCL	ULCL	Percentage of total effect
Total effect	Direct Path	−0.268	0.030	−0.326	−0.209	100.00
Direct effect		−0.178	0.031	−0.238	−0.117	66.42
Total indirect effect		−0.090	0.013	−0.117	−0.065	33.58
Indirect effect	Pathway 1	−0.043	0.010	−0.062	−0.025	16.04
	Pathway 2	−0.041	0.010	−0.061	−0.024	15.30
	Pathway 3	−0.006	0.002	−0.011	−0.003	1.24

**Figure 2:**
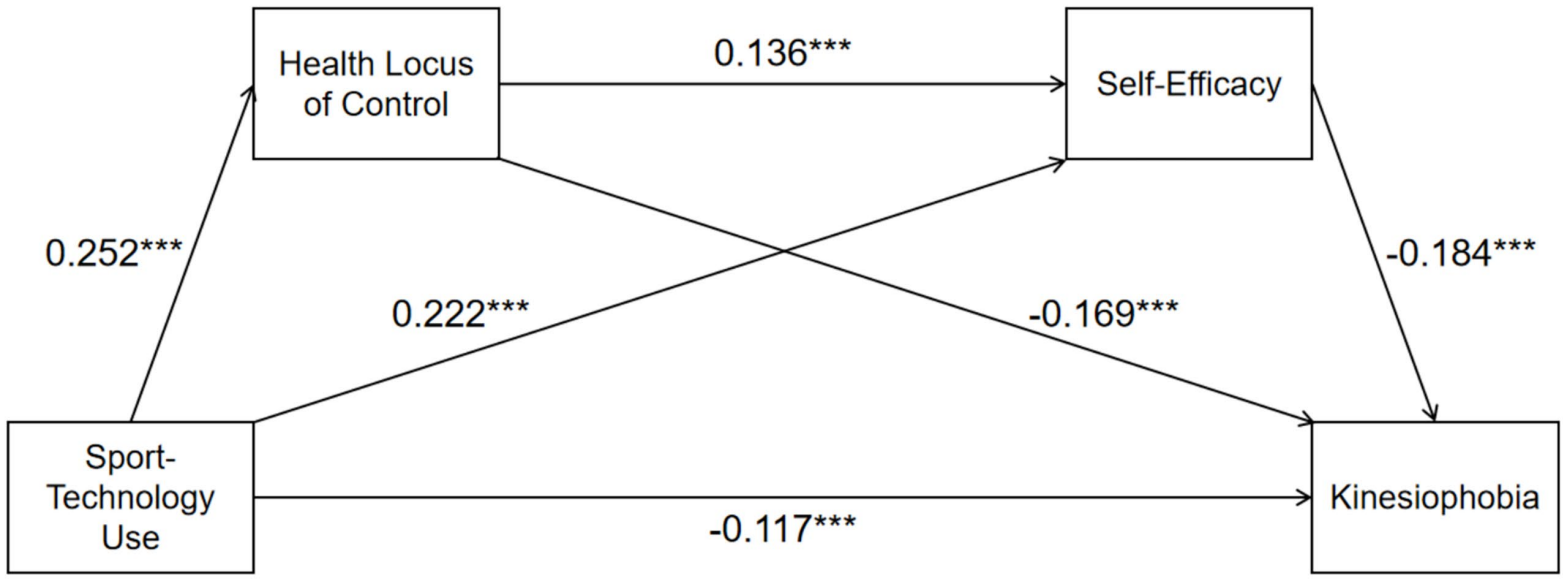
Structural path model.

With respect to specific indirect pathways, Indirect Path 1 (sport-technology use → health locus of control → kinesiophobia) was significant (β = −0.043, 95% CI [−0.062, −0.025]), accounting for 16.04% of the total effect. Indirect Path 2 (sport-technology use → self-efficacy → kinesiophobia) was also significant (β = −0.041, 95% CI [−0.061, −0.024]), accounting for 15.30% of the total effect. The magnitudes of these two indirect effects were comparable, suggesting that health locus of control and self-efficacy contributed similarly when acting as independent mediators. Indirect Path 3, representing the serial mediation pathway (sport-technology use → health locus of control → self-efficacy → kinesiophobia), was statistically significant but substantially smaller in magnitude (β = −0.006, 95% CI [−0.011, −0.003]), accounting for only 1.24% of the total effect. In contrast, the two independent mediation pathways (Path 1 and Path 2) each explained over 15% of the total effect, collectively representing the vast majority of the indirect association. While this result supports the theoretical plausibility of a serial transmission mechanism, its practical significance is considerably weaker than that of the two independent pathways (see Figure 2).

Overall, the hypothesized mediation model was supported. Sport-technology use was not only directly linked to lower kinesiophobia but also exerted indirect effects through both independent and serial mediation pathways involving health locus of control and self-efficacy. These findings deepen our understanding of the psychological mechanisms through which sport-technology products may facilitate physical activity participation among people with disabilities and provide empirical evidence to inform intervention strategies.

## 5 Discussion

In the present study, sport-technology use was conceptualized as a modifiable external resource within research on psychological barriers to physical activity participation among people with disabilities. By specifying a serial mediation pathway through health locus of control and self-efficacy, the findings provide both theoretical relevance and practical explanatory value. From an integrative theoretical perspective, the fear–avoidance model posits that when individuals face bodily discomfort or potential harm, catastrophic interpretations and threat sensitivity may trigger avoidance. Avoidance then be associated with lower levels of activity, deconditioning, and functional limitations, which in turn reinforce fear and uncertainty (Vlaeyen and Linton, 2000). Due to functional constraints and prior experiences of exercise-related setbacks, people with disabilities may be particularly likely to develop a cognitive representation of exercise contexts as “high risk–low controllability.” The real-time monitoring, risk alerts, and corrective feedback provided by sport-technology products can be statistically interpreted as consistent with a mechanism of “making threat cues transparent.” On the one hand, such functions can reduce ambiguity during exercise by transforming risk from something “indescribable” into something “identifiable and manageable.” On the other hand, immediate feedback and staged goals can break exercise tasks into achievable units, facilitating a behavioral shift from avoidance to approach. Accordingly, it is not surprising that the direct effect accounted for a larger proportion of the total effect. This pattern suggests that technological tools may first enable a context-level recoding of risk before belief change occurs, thereby rapidly reducing the intensity of kinesiophobia (i.e., fear of movement); belief-based pathways may then serve primarily as consolidating and maintenance functions, determining whether decreases in fear translate into sustained physical activity engagement.

Crucially, the present study measured sport-technology use as a composite index of overall usage intensity (behavioral frequency, functional depth, and sustained dependence) without distinguishing between different types of tools (e.g., wearables, feedback systems, rehabilitation devices, mobile apps). Therefore, our findings demonstrate a statistical association between the global pattern of sport-technology engagement and lower kinesiophobia, but they do not permit causal attribution to any specific technological feature or design element.

The mediating pathway of health locus of control and self-efficacy provides a testable psychological explanation for this coupling of “external tools” and “internal resources.” Self-efficacy theory emphasizes that individuals’ judgments about their capability to perform specific behaviors directly shape goal setting, persistence, and emotional responses; when self-efficacy increases, difficulties are more likely to be construed as solvable tasks, and anxiety and fear tend to decrease. In health promotion contexts, self-efficacy is also viewed as a core pathway of behavior change, forming a self-regulatory structure together with outcome expectancies, goals, and environmental facilitators (Bandura, 2004). Consistent with the present findings, the independent mediating contribution of self-efficacy was comparable to that of health locus of control, indicating that in the emergence and alleviation of kinesiophobia among people with disabilities, two cognitive anchors—“Can I do it?” and “Can I control the outcome?”—may be equally critical. By recording progress, visualizing training load, and providing error-correction information, the use of sport-technology products can be positively correlated with mastery experiences, the most influential source of efficacy beliefs, enabling users to accumulate successful experiences at lower cost and attenuate fear responses. At the same time, technology-based feedback can clarify the causal chain between exercise behavior and health outcomes, shifting perceived health results from “external chance” to “behaviorally malleable,” thereby enhancing health locus of control.

It is critical to first acknowledge that the serial indirect effect, while statistically significant, accounted for only 1.24% of the total effect—a magnitude that is considerably smaller than the two independent pathways (each >15%). Therefore, its theoretical importance should be interpreted cautiously and considered as supplementary rather than central to the overall model. With this caveat, the serial pathway does offer a plausible, albeit minor, additional mechanism: a sequential chain of “control beliefs first → capability beliefs next → lower kinesiophobia.” However, the dominant explanatory weight lies clearly with the independent mediating roles of health locus of control and self-efficacy. That is, when people with disabilities first develop a sense that health outcomes are controllable, they may be more likely to perceive exercise as a manageable process, become willing to invest effort, and subsequently develop stronger self-efficacy, ultimately reducing kinesiophobia. This sequence aligns closely with feedback-loop accounts of self-regulation. Self-regulation depends on iterative cycles of “goal–feedback–correction,” in which clear standards and actionable feedback are negatively associated with helplessness arising from perceived goal discrepancies and promote sustained action. Sport-technology products provide high-frequency feedback and discrepancy cues, embedding “control” not as an abstract belief but as a feature of the micro-processes of everyday physical activity. Given its small magnitude, the serial effect should not be overemphasized. Nevertheless, it tentatively suggests two possibilities (while acknowledging that these are secondary to the independent pathways): First, health locus of control and self-efficacy may partially overlap functionally; particularly in health behavior contexts, both may share a core of perceived agency, limiting the incremental explanatory value of a strict sequence. Second, the key pathways through which sport-technology products reduce fear may not rely exclusively on these sequential beliefs, as evidenced by the much larger direct effect and independent indirect effects. Other mechanisms not included in the current model may also be influential, such as calibration of risk perception, emotion regulation strategies, availability of social support, or online guidance from rehabilitation professionals. This interpretation is consistent with integrative behavior change frameworks, which emphasize that behavioral outcomes typically arise from the coupled system of capability, opportunity, and motivation; No single psychological construct can fully account for behavior (Michie et al., 2011).

Because the present study did not compare different types of sport-technology products (e.g., wearables, feedback systems, rehabilitation devices, mobile apps), the following interpretations regarding specific product features are speculative and are offered solely as hypotheses to guide future research, not as conclusions directly supported by our data.

From a practical standpoint, the findings suggest that sport-technology use is associated with lower kinesiophobia partly through enhanced health locus of control and self-efficacy. Therefore, to maximize the psychological benefits, intervention programs for people with disabilities could emphasize two components: (1) functions that help users perceive clear control over their health outcomes (e.g., visualized progress feedback, goal-setting tools); and (2) features that accumulate mastery experiences (e.g., graded exercise tasks, positive reinforcement). These components directly target the two independent mediating pathways identified in this study, rather than relying on untested technological features or policy measures.

## 6 Conclusions and future directions

### 6.1 Conclusion

Sport-technology use was negatively associated with kinesiophobia in this sample among our sample, and this association was statistically explained by independent and serial indirect associations via health locus of control and self-efficacy. However, it is paramount to interpret these findings within the context of the study’s sample. As detailed in the Methods section, our participants represent a specific, potentially more motivated and digitally-engaged sub-group of people with disabilities. The following discussion, therefore, applies to this sub-population, and theoretical and practical implications for the broader disabled community are suggestive rather than definitive.

(1) Sport-technology use significantly and was negatively correlated with kinesiophobia. That is, higher intensity of sport-technology use was associated with lower levels of kinesiophobia among people with disabilities, providing empirical support for the practical value of sport-technology products in alleviating exercise-related fear in this population.

(2) Health locus of control played a significant independent mediating correlate between sport-technology use and kinesiophobia. Sport-technology use exhibited a positive correlation with health locus of control, and stronger health locus of control further predicted lower kinesiophobia, offering an important explanatory mechanism for the relationship.

(3) Self-efficacy also showed a significant independent mediating effect. By lowering participation barriers and providing positive feedback, Sport-technology use was positively correlated with self-efficacy, which in turn effectively attenuated kinesiophobia.

(4) Health locus of control and self-efficacy also exhibited a statistically significant but very modest serial mediation effect, accounting for only 1.24% of the total effect—an order of magnitude smaller than the two independent pathways (each >15%). Therefore, this serial pathway should be viewed as a minor, supplementary mechanism rather than a primary explanatory route. While it provides an additional lens for understanding sequential cognitive processes, its theoretical and practical weight is substantially lower than that of the two independent mediating pathways.

In summary, the association between sport-technology use and kinesiophobia among people with disabilities operates through both a direct pathway and indirect pathways via independent and serial mediation through health locus of control and self-efficacy. Together, these pathways delineate a coherent associative pattern linking “use behavior, psychological resources, and emotional outcome.”

### 6.2 Limitations

Although this study systematically explored the mechanism through which sport-technology use relates to kinesiophobia among people with disabilities, several limitations should be addressed in future research:

(1) Limited generalizability due to specific inclusion criteria. The most significant limitation is the restricted nature of the sample. By design, we included only individuals who were active users of sport-technology (≥1 month) and physically active (past 6 months), while excluding those with intellectual disabilities, multiple disabilities, and those who were inactive or non-users of technology. Consequently, our findings directly inform our understanding of active, technology-using adults with physical, visual, or hearing impairments. They do not, and cannot, speak to the experiences of the broader, heterogeneous population of people with disabilities, especially those who are the most sedentary, have the most severe functional limitations, or lack access to or interest in digital health tools. Future research must intentionally recruit from these excluded groups to test whether the observed serial mediation model holds, is attenuated, or is entirely absent.

(2) Constraints of research design and causal inference. The cross-sectional design can reveal associations among variables but cannot establish causal directions among sport-technology use, health locus of control, self-efficacy, and kinesiophobia, nor can it capture dynamic changes over time. In addition, all measures relied on self-report questionnaires; the absence of objective behavioral indicators or physiological measures may have introduced response bias.

(3) Limited scope of variables and analytical depth. The model focused on the serial mediation pathway of health locus of control and self-efficacy but did not include potential moderators (e.g., social support, duration of disability, or exercise type) or explore subgroup differences across disability severity or age groups. As a result, boundary conditions of the observed relationships may have been overlooked. Furthermore, sport-technology products were not differentiated by type; instead, use intensity was assessed broadly, making it difficult to identify potentially distinct mechanisms across different product categories.

(4) Lack of contextual and longitudinal considerations. The study did not account for differences in accessibility and use contexts (e.g., urban vs. rural environments), which may influence sport-technology adoption and its effects. In addition, the absence of longitudinal follow-up precludes evaluation of the long-term stability of the relationships and prevents comparisons between short-term and sustained use, limiting insights into the dynamic evolution of the effect of sport-technology use on kinesiophobia.

(5) Several limitations of this study warrant mention, particularly regarding the exploration of alternative relational models and divergent causal orientations. Our cross-sectional design cannot definitively establish causal relationships between variables, and we acknowledge the possibility of reverse causality: individuals with lower levels of kinesiophobia may have higher willingness and capacity to use sport-technology products, which may in turn further strengthen their health locus of control and self-efficacy.

(6) Construct mismatch between the self-efficacy measure and the outcome. The present study used a general self-efficacy scale rather than an exercise- or sport-specific measure. Given that kinesiophobia is inherently tied to movement and physical activity, this difference in specificity may have introduced construct-level imprecision. Although a general measure was chosen for theoretical consistency with the domain-general health locus of control and for cross-disability measurement equivalence, it does not fully capture an individual’s confidence in performing exercise-related tasks. Consequently, the mediating role of self-efficacy may be conservatively estimated or misestimated, and our findings cannot be directly generalized to exercise-specific self-efficacy. Future research should replicate the model using a validated exercise-specific self-efficacy scale for people with disabilities.

### 6.3 Future directions

From a theoretical perspective, future research could further expand the scope and depth of existing models. While the present study confirmed the serial mediation of health locus of control and self-efficacy, subsequent work may incorporate potential moderators to enhance explanatory power. For example, differences in functional limitations across disability types, the level of technological iteration of sport-technology products, and structural characteristics of social support networks may moderate both the direct association between sport-technology use and kinesiophobia and the proposed serial mediation pathways. Examining such moderation effects would help clarify the boundary conditions and contextual applicability of the theoretical framework. Moreover, cross-sectional designs are limited in their ability to reveal long-term dynamic and causal relationships. Future studies could adopt longitudinal designs with multi-wave data collection to examine how trajectories of sport-technology use influence the long-term development of health locus of control, self-efficacy, and kinesiophobia, thereby testing the stability and temporal validity of the serial mediation effect and providing stronger evidence for causal inference. In addition, integrating multi-disciplinary theoretical perspectives—such as social cognitive theory and self-determination theory—may enable a deeper investigation of the internal cognitive processes through which sport-technology use meets basic psychological needs (autonomy, competence, and relatedness) and subsequently shapes health locus of control and self-efficacy. Such integration may move beyond the limitations of a single-theory lens and contribute to a more systematic and comprehensive explanatory framework. Finally, future research may extend outcomes beyond kinesiophobia to related endpoints, such as sustained physical activity participation, tendencies toward exercise addiction, and subjective health-related well-being. Thereby establishing a more complete “antecedents–mediators–outcomes” chain and enriching the interdisciplinary field at the intersection of disability sport psychology and sport technology.

From a practical perspective, the findings directly support two intervention targets for reducing kinesiophobia among active sport-technology users with disabilities. First, to enhance health locus of control, interventions could incorporate features that make health outcomes more observable and personally controllable, such as real-time feedback on exercise performance or progress tracking. Second, to strengthen self-efficacy, graded task settings and positive reinforcement for achieved goals should be embedded. These two targets correspond to the independent mediating pathways identified in this study, which together accounted for the majority of the indirect effect. The serial pathway, while statistically significant, had a very small effect size and should be considered a secondary, long-term focus rather than a primary intervention target. Future research could test whether these two psychological mechanisms can be experimentally manipulated through specific design elements of sport-technology products, using controlled trials rather than cross-sectional designs. By simulating realistic exercise contexts and providing immersive physical activity experiences, Such products may further improve usability and attractiveness, ultimately enhancing the quality of physical activity participation and supporting continued progress toward social inclusion for people with disabilities.

While our study focused on the serial mediation of health locus of control and self-efficacy, we recognize that there are other divergent relational frameworks that may explain the association between sport-technology use and kinesiophobia. For example, moderated mediation models incorporating variables such as disability type, functional impairment severity, age, and previous exercise experience may reveal boundary conditions of the proposed effects; in addition, other mediating variables such as social support, body image, and exercise motivation may also play a role in this association, which could be explored in future research to expand the explanatory framework of kinesiophobia among people with disabilities.

## Data Availability

The original contributions presented in the study are included in the article/Supplementary material, further inquiries can be directed to the corresponding author.
